# Prevalence and risk factors of some arthropod-transmitted diseases in cattle and sheep in Jordan

**DOI:** 10.14202/vetworld.2020.201-205

**Published:** 2020-01-28

**Authors:** Zaidoun S. Hijazeen, Zuhair Bani Ismail, Ahmad M. Al-Majali

**Affiliations:** Department of Veterinary Clinical Sciences, Faculty of Veterinary Medicine, Jordan University of Science and Technology, Irbid 22110, Jordan

**Keywords:** arthropod-transmitted diseases, biosecurity, control measures, livestock, ruminants

## Abstract

**Aims:**

The objectives of this study were to determine the prevalence and associated risk factors of bluetongue virus (BTV) in sheep and bovine ephemeral fever virus (BEFV) in dairy cattle in Jordan.

**Materials and Methods:**

A simple randomized study was designed to collect 600 serum samples from sheep and 300 serum samples from dairy cattle located in the Northwestern parts of Jordan. In addition, data regarding farm management were collected using a pre-tested questionnaire through personal interview to determine potential risk factors. The seroprevalences of BEF and BTVs were determined using serum neutralization test and BTV group-specific competitive enzyme-linked immunosorbent assay, respectively.

**Results:**

The overall seroprevalence of neutralizing antibodies against BEFV in dairy cattle was 45.37%. The overall seroprevalence of BTV group-specific antibodies in sheep was 47.8% (54% true seroprevalence). Logistic regression analysis identified geographic location (Irbid) (odds ratio [OR]=1.0; confidence interval [CI]=0.5-2.1), no use of disinfectants on the farm (OR=1.0; CI=0.05-0.1), and lack of veterinary services (OR=10; CI=3.5-13.2) as risk factors associated with high seropositivity against BTV in sheep. Geographic location (Jarash) (OR=3; CI=1.0-5.5), age of the animal (1-2 years of age (OR=1; CI=0.3-1.9), and lack of veterinary services (OR=9; CI=4-11) were identified as risk factors associated with high seroprevalence against BEFV in dairy cattle.

**Conclusion:**

Results of this study indicate that BEFV in dairy cattle and BTV in sheep are endemic in Northwestern regions of Jordan. Implementation of appropriate control measures is, therefore, required to reduce the adverse effects of these diseases on animal health and productivity.

## Introduction

Arthropod-transmitted viruses in ruminants including the bluetongue virus (BTV) and bovine ephemeral fever virus (BEFV) are important diseases that may result in significant economic losses [1-4]. Arthropod-transmitted viral diseases of livestock animals are associated with severe economic losses due to reduced animal productivity, costs of veterinary services, and restrictions on trade and transboundary transportation of animals and animal products [5,6].

The BTV belongs to the genus *Orbivirus* of the *Reoviridae* family. The virus is transmitted by bites of *Culicoides* midges [7]. Bluetongue disease is considered endemic in many parts of the world including Africa, Asia, and North and South America. Most affected cattle are asymptomatic and act as a natural reservoir of BTV [8]. In sheep, peracute and acute clinical signs of BTV infection are characterized by respiratory dyspnea, swollen tongue, and high morbidity and mortality [9]. BEF is considered a non-contagious viral disease of cattle and water buffalo [1]. It is widely spread in many tropical and subtropical regions in Africa, Asia, Australia, and the Middle East [10,11]. The virus belongs to the *Ephemerovirus* genus of the *Rhabdoviridae* family. The virus is likely transmitted by bites of mosquitoes and midges [10,11]. Clinically, the disease is characterized by high fever, lymphadenopathy, oculonasal discharge, subcutaneous edema, respiratory distress, muscle tremors and stiffness, lameness, and eventually recumbency [12].

Proper understanding of the epidemiology, transmission, and risk factors of arthropod-transmitted diseases is mandatory to develop and implement effective control and prevention measures of these diseases. In Jordan, no reports could be cited in the literature concerning the prevalence of BTV and BEF in livestock populations.

Therefore, the objectives of this study were to determine the prevalence and associated risk factors of BTV in sheep and BEF in dairy cattle.

## Materials and Methods

### Ethical approval and informed consent

All procedures performed in this study were reviewed and approved by the Institutional Animal Use and Care Committee of Jordan University of Science and Technology. Written and signed consents were obtained from farm owners before the study was conducted.

### Study area

Dairy cattle and sheep production are mainly concentrated in the Northwestern regions of Jordan due to suitable weather and availability of water sources [13,14]. Jordan enjoys long moderate to hot and dry summers that extend from mid-April to mid-October while winters are short and cold and extend from December to February [14]. The annual rainfalls in the Northwestern regions of Jordan range from 100 mm to 700 mm [14]. Animal production systems are mostly small house holdings to medium-large semi-intensive [13]. Concentrate feed supplement is usually provided most of the year [13].

### Selection criteria and sample size

This study was conducted during the spring and summer of 2012. The targeted municipalities located in Northwestern Jordan were Mafraq, Irbid, Jarash, Ajloun, and Northern part of Jordan Valley. Farms within each municipality were selected using a simple random sampling method. The sample size for each area was calculated according to the equation: N=(1−α^1/D^)×(N−[D−1/2]); where α is 1 – confidence, N is the population size, and D is the expected number of diseased animals in the population [15]. Accordingly, 300 blood samples from dairy cows and 600 blood samples from sheep were collected randomly from selected farms.

### Blood samples collection

Whole blood samples were collected through jugular vein puncture using hypodermic needles and plain Vacutainer tubes. Samples were transferred to the laboratory on ice within 3-4 h after collection. Serum was collected by centrifugation of clotted whole blood samples at 3000 g for 10 min. Serum was stored at −80°C until analysis was performed.

### Serum neutralizing antibodies

Serum samples from dairy cattle were subjected to serum neutralization test (SNT) to detect antibodies against BEFV according to previously published methods [6]. Briefly, serum samples were inactivated first at 56°C for 30 min. Inactivated serum samples were then subjected to a 2-fold dilution (1:4-1:512) in 96-well microplates using minimum essential medium cell culture media supplemented with antibiotics and fetal calf serum (Merck, USA). To perform the serial dilution, 75 µl of medium were added to first well while the rest of the wells received 50 µl of medium. Then to the first well, 25 µl of sample was added followed by transferring 50 µl into the next well. To each well, 50 µl of OIE standard reference BEF strain (TCID_50_; Onderstepoort Veterinary Institute, South Africa) were added and the plates were incubated for 60 min at 37°C and 5% CO_2_. After that, 150 µl of VERO cells were added to each well and the plates were incubated again for 2-3 days at 37°C and 5% CO_2_. The plates were then read by observing the presence of distinctive cytopathic effects (CPEs). The highest serum titer was determined as the concentration at which more than 75% of CPEs were inhibited.

### Complement enzyme-linked immunosorbent assay (cELISA)

Serum samples from sheep were analyzed using BTV-specific cELISA kits according to the manufacturer’s instructions (IDEXX, France). Positive and negative controls were provided by the manufacturer. The microplates were read by measuring the optical density at 450 nm.

### Questionnaire

A pre-tested questionnaire to collect data related to management, health, and production was administered by personal interview of the owners or managers of participating farms. Data collected included geographic location of the farm, type of management system used on the farm, herd size, age of the animals, presence of farm dogs, use of disinfectants on the farm, purchasing of new animals from local markets, availability of regular veterinary services, sources of drinking water, and sources of feedstuff.

### Statistical analysis

Risk factors were evaluated by transforming the collected data into Microsoft Excel sheets. Data were then subjected to initial screening using Chi-square test (univariate). Parameters with significant differences were then subjected to multivariate logistic regression model. Risk factors were considered statistically significant at p≤0.05. Statistical analysis was performed using SPSS software version 24 (IBM, NY, USA).

## Results

### Seroprevalence of neutralizing antibodies against BEFV in dairy cattle

The overall seroprevalence of neutralizing antibodies against BEFV in dairy cattle in the study area was 45.37%. The seroprevalences according to the geographical location were 56.7%, and 36.9% in Jarash and Jordan Valley, respectively. No positive samples were detected in Irbid, Mafraq, and Ajloun. The serum titers of neutralizing antibodies ranged from 1:4 to 1:64.

### Seroprevalence of BTV in sheep

The overall seroprevalence of BTV serogroup-specific antibodies in sheep in the study area was 47.8%. The true seroprevalence was 54% based on 99.1% specificity, 84.5% sensitivity, and 84.5% of the ELISA test. The individual animal seroprevalence within positive flocks ranged from 25.5% to 70.7%. According to the geographic location of the study region, the seroprevalences were 70.0%, 44.7%, 45.1%, 56.4%, and 25.5% in Irbid, Mafraq, Jordan Valley, Ajloun, and Jarash, respectively.

### Risk factors

Results of risk factor analysis for BTV infection in sheep and BEF infection in cattle are presented in Tables-1-3. Logistic regression analysis identified geographic location (Irbid) (odds ratio [OR]=1.0; confidence interval [CI]=0.5-2.1), no use of disinfectants on the farm (OR=1.0; CI=0.05-0.1), and lack of veterinary services (OR=10; CI=3.5-13.2) as risk factors associated with high seropositivity against BTV in sheep. In cattle, logistic regression analysis identified geographic location (Jarash) (OR=3; CI=1.0-5.5), age of the animal (1-2 years of age (OR=1; CI=0.3-1.9), and lack of veterinary services (OR=9; CI=4-11) as risk factors associated with high seroprevalence against BEFV in dairy cattle.

**Table-1:** Univariate analysis of risk factors associated with high seropositivity of bluetongue virus group-specific antibodies in sheep (n=600).

Risk factor	Category	Seropositivity, n (%)
Geographic location	Mafraq	89 (44.7)
	Jordan Valley	51 (43.6)
	Ajloun	53 (56.3)*
	Irbid	70 (70.0)
	Jarash	24 (56.4)
Age	1-2 years	113 (55.7)
	3-5 years	174 (43.8)
Presence of farm dogs	Yes	198 (49.1)
	No	89 (45.2)
Use of disinfectants	Yes	166 (33.1)*
	No	121 (56.3)
Purchasing of new animals	Yes	198 (49.5)
	No	89 (44.5)
Availability of veterinary services	Yes	102 (28.3)*
	No	145 (47.4)

* p≤0.05

**Table-2:** Univariate analysis of risk factors associated with high seropositivity against bovine ephemeral fever virus in dairy cattle (n=108).

Risk factors	Category	Seropositivity, n (%)
Geographic location	Jarash	25 (76.6)*
	Jordan Valley	25 (38.5)
Type of farm	Cattle	36 (40.9)
	Mixed cattle and sheep	14 (70)
Age	1-4 years	36 (75)*
	4>years	14 (20)
Herd size	1-49	24 (48)
	50-299	10 (31.3)
	<300	16 (61.5)
Sources of drinking water	Municipality	14 (53.8)
	Others	36 (43.9)
Sources of feedstuff	Readily mixed	0 (0)
	Mixing in the farm	50 (49)
Presence of farm dogs	Yes	25 (58.1)
	No	25 (38.5)
Purchasing of new animals	Yes	1 (6.7)
	No	49 (52.7)
Availability of veterinary services	Yes	11 (24.4)
	No	39 (61.9)*

* p≤0.05

**Table-3:** Multivariate logistic regression analysis of risk factors associated with high seropositivity against bluetongue virus in sheep and bovine ephemeral fever virus in dairy cattle.

Risk factors	OR	95% confidence interval odds ratio	p-value
Bluetongue virus in sheep			
Geographic location (Irbid)	1.0	0.4-0.7	0.05
No use of disinfectants on the farm	1.0	0.05-0.1	0.05
Lack of veterinary services	10.0	3.5-13.2	0.001
Bovine ephemeral fever virus in dairy cattle			
Geographic location (Jarash)	3	1.0-5.5	0.05
1-2 years of age	1.0	0.03-0.09	0.05
Lack of veterinary services	9.0	4-11	0.001

## Discussion

BEF and BTVs are economically important viral diseases that affect a wide range of domestic and wild ruminants throughout the world [16,17]. In this study, the diagnosis of BTV was accomplished using cELISA which is a serogroup-specific assay able to measure BTV antibodies without the cross-reactivity problems of other tests like agar gel immunodiffusion test. Moreover, cELISA is the prescribed test for international trade by the OIE terrestrial manual and is extensively used worldwide [18]. BEF is a rare disease in Jordan, and therefore, SNT was used according to the OIE recommendations which stated that “single SNT-positive serum sample may be suggestive of BEF in areas where this disease does not normally occur” [19].

This is the first study in Jordan that indicated the presence of BEFV in dairy cattle and BTV in sheep. In this study, the seroprevalence of BEFV and BTV was 45.37% and 47.8%, respectively. These results are in agreement with previously reported data that indicate widespread of BTV in most countries lying in the tropics and subtropics primarily between latitudes 40°N and 35°NS [17]. In the neighboring countries, the seroprevalence of BTV has been reported around 34% in Iran [20,21], 54.1% in Saudi Arabia [22], and 29.5% in Turkey [23]. Although no clinical signs of BTV infection in sheep were detected during the collection of samples from selected animals, the high seroprevalence of the disease in the targeted population indicates previous exposure, especially no vaccination programs are implemented in this region. This suggests that the vector responsible for BTV transmission is available and reemergence of the disease could arise in the future; therefore, appropriate control and prevention measures such as biosecurity and vaccination programs must be implemented.

The overall seroprevalence of BEF in dairy cattle in Northwestern region of Jordan was 45.37%. The highest prevalence of BEF (56.7%) was reported in Jarash municipality. No vaccination program against BEF is in effect in Jordan and the cattle population is highly susceptible to this disease. The widespread of antibodies against BEF in Jordan indicates previous natural exposure to the virus. This virus may be introduced into Jordan from importation of live cattle from endemic countries or incursions of BEFV from adjacent countries as several outbreaks occurred in Israel [24], Saudi Arabia [25], and Egypt [26]. It has been reported that BEFV can be transmitted to new territories through winds and animal transportation [16].

In this study, risk factor analysis identified the geographic location of the farm, not using disinfectants on the farm and lack of regular veterinary services as risk factors for seropositivity of BTV in sheep. Similar observations were reported in other parts of the world, in which the location of the farm affected significantly the seroprevalence of the disease [27]. It has been reported that arthropod-borne diseases are affected by season and geographic location. This could be due to different climates, altitudes, and wind activity which, in turn, affects *Culicoides* activity in the region [21]. It is obvious that lack of regular use of disinfectants and absence of veterinary services in farms resulted in widespread of BTV. It is obvious that providing professional veterinary care to diseased animals results in improved outcomes and decreases morbidity and mortality rates. Risk factor analysis revealed the geographic location, age of the animal, and lack of regular veterinary services as risk factors of BEF infection in dairy cattle. These results are similar to previously reported findings of arthropod-borne viruses [21,25]. It has been reported that the region, breed, sex, and age have a significant effect on the seroprevalence of BEFV [25]. In general, young cattle (1-2 years of age) are more susceptible to BEF due to low immunity. In addition, there is no vaccination program against BEF in place in Jordan.

## Conclusion

The results of this study indicate that BEFV in dairy cattle and BTV in sheep are endemic in Northwestern regions of Jordan. Implementation of appropriate control measures is, therefore, required to reduce the adverse effects of these diseases on animal health and productivity in Jordan.

## Authors’ Contributions

ZSH conceived, conducted the fieldwork, administered the questionnaires, and drafted the manuscript. ZBI conducted the literature search, data interpretation, and edited the manuscript. AMA designed and supervised the study, performed the statistical analysis, and reviewed the manuscript. All authors read and approved the final manuscript.
